# *Lactobacillus delbrueckii* CIDCA 133 Ameliorates Chemotherapy-Induced Mucositis by Modulating Epithelial Barrier and TLR2/4/Myd88/NF-κB Signaling Pathway

**DOI:** 10.3389/fmicb.2022.858036

**Published:** 2022-04-26

**Authors:** Fernanda Alvarenga Lima Barroso, Luís Cláudio Lima de Jesus, Tales Fernando da Silva, Viviane Lima Batista, Juliana Laguna, Nina Dias Coelho-Rocha, Kátia Duarte Vital, Simone Odília Antunes Fernandes, Valbert Nascimento Cardoso, Enio Ferreira, Flaviano Santos Martins, Mariana Martins Drumond, Pamela Mancha-Agresti, Alexander Birbrair, Debmalya Barh, Vasco Azevedo

**Affiliations:** ^1^Departamento de Genética, Ecologia e Evolução, Universidade Federal de Minas Gerais, Belo Horizonte, Brazil; ^2^Departamento de Análises Clínicas e Toxicológicas, Universidade Federal de Minas Gerais, Belo Horizonte, Brazil; ^3^Departamento de Patologia Geral, Universidade Federal de Minas Gerais, Belo Horizonte, Brazil; ^4^Departamento de Microbiologia, Universidade Federal de Minas Gerais, Belo Horizonte, Brazil; ^5^Departamento de Ciências Biológicas, Centro Federal de Educação Tecnológica de Minas Gerais, Belo Horizonte, Brazil; ^6^Centre for Genomics and Applied Gene Technology, Institute of Integrative Omics and Applied Biotechnology (IIOAB), Purba Medinipur, India

**Keywords:** 5-FU-induced mucositis, probiotics, *Lactobacillus delbrueckii*, anti-inflammatory cytokines, intestinal permeability, tight junction proteins

## Abstract

Intestinal mucositis promoted by the use of anticancer drugs is characterized by ulcerative inflammation of the intestinal mucosa, a debilitating side effect in cancer patients undergoing treatment. Probiotics are a potential therapeutic option to alleviate intestinal mucositis due to their effects on epithelial barrier integrity and anti-inflammatory modulation. This study investigated the health-promoting impact of *Lactobacillus delbrueckii* CIDCA 133 in modulating inflammatory and epithelial barrier markers to protect the intestinal mucosa from 5-fluorouracil-induced epithelial damage. *L. delbrueckii* CIDCA 133 consumption ameliorated small intestine shortening, inflammatory cell infiltration, intestinal permeability, villus atrophy, and goblet cell count, improving the intestinal mucosa architecture and its function in treated mice. Upregulation of *Muc2*, *Cldn1*, *Hp*, *F11r*, and *Il10*, and downregulation of markers involved in NF-κB signaling pathway activation (*Tlr2, Tlr4, Nfkb1, Il6*, and *Il1b*) were observed at the mRNA level. This work suggests a beneficial role of *L. delbrueckii* strain CIDCA 133 on intestinal damage induced by 5-FU chemotherapy through modulation of inflammatory pathways and improvement of epithelial barrier function.

## Introduction

Intestinal mucositis is a commonly reported side effect in oncology patients undergoing chemoradiotherapy (Sonis, 2004; Kim et al., 2018) due to non-selective anticancer drugs (Miura et al., 2010; Crombie and Longo, 2016). 5-Fluorouracil (5-FU), an antimetabolite analog to uracil, is one of the most frequently used antitumor drugs that cause mucositis, with an incidence higher than 40% of cancer cases even if treated with the standard dose (10-15 mg/kg for 3-4 days intravenously). 5-FU exerts cytotoxic effects by inhibiting thymidylate synthase (TS) and incorporating its metabolites into DNA/RNA, leading to cell death and apoptosis (Longley et al., 2003; Miura et al., 2010). However, due to its non-selectivity, besides affecting neoplastic cells, this drug also promotes damage to healthy cells (Miura et al., 2010; Crombie and Longo, 2016).

Mucositis caused by 5-FU affects the small bowel mucosa, characterized by intense inflammation, dysbiosis, alteration in intestinal epithelium architecture such as villus atrophy, and loss of epithelial barrier integrity due to tight junction protein disruption and goblet cell degeneration. Additionally, it subsequently increases intestinal permeability and reduces mucin secretion (Sonis, 2004; van Vliet et al., 2010; Song et al., 2013). Except for palliative therapy, there is no known treatment option to prevent chemotherapy-induced intestinal mucositis (Batista et al., 2020).

Probiotic strains, such as *Lactobacillus* spp., mainly belong to the Lactic Acid Bacteria group (LAB), and they have been investigated as an alternative therapeutic approach against intestinal mucositis (Batista et al., 2020). Several studies have shown the efficacy of various LABs in preventing intestinal mucositis by regulating the microbiota in dysbiosis (Chang et al., 2018), improving the inflammatory process, modulating oxidative stress (Justino et al., 2015; Hu et al., 2020; Quaresma et al., 2020), and protecting the epithelial barrier by maintaining the integrity of tight junction proteins and goblet cells (Yeung et al., 2015; Barroso et al., 2021). Despite these beneficial effects, the use of these inactivated microorganisms has also been highlighted (Jin et al., 2020; Nakai et al., 2021; Trindade et al., 2021), to minimize possible risks of bacteria translocation producing systemic infections, especially in premature infants or immune-compromised patients (Doron and Snydman, 2015; Zawistowska-Rojek and Tyski, 2018). In addition, it has been demonstrated that many of the effects (such as immunomodulation and microbiota regulation) of inactivated probiotics are similar to their metabolically active form, suggesting that these effects can be attributed to cell wall proteins of these microorganisms that interact with host cells during their passage through the gastrointestinal tract (GIT) (Hsieh et al., 2016; Aiba et al., 2017; Jin et al., 2020; Nobre et al., 2022).

Among the *Lactobacillus* genus, *Lactobacillus delbrueckii* subsp. *lactis* CIDCA 133, a strain isolated from raw cow’s milk, has been related to probiotic properties. This includes its ability to inhibit the growth of spoilage bacteria, such as *Escherichia coli* and *Pseudomonas aeruginosa* (Kociubinski et al., 1999; Hugo et al., 2008), and to stimulate dendritic cells and murine macrophages infected with pathogenic *Bacillus cereus* or *Citrobacter rodentium* (Rolny et al., 2016; Hugo et al., 2017). One of our pioneering studies showed that a fermented milk formulation containing *L. delbrueckii* CIDCA 133 could attenuate antineoplastic 5-FU-induced intestinal epithelial tissue disruption by inhibiting degeneration of goblet cells, intestinal permeability, and inflammatory cell infiltration (de Jesus et al., 2019). In our other studies, we reported that no adverse effect was observed in healthy BALB/c mice after CIDCA 133 consumption, but its consumption stimulated the mucosal immune system and epithelial barrier by upregulating the gene expression of tight junction protein occludin (*Ocln*) (de Jesus et al., 2021a), anti-inflammatory cytokines (*Il10* and *Tgfb1*) and mucin 2 (*Muc2)*, and inhibiting the proinflammatory transcription factor *Nfkb1* (p105) (de Jesus et al., 2021b). In this context, this study investigated whether the modulation of inflammatory and epithelial barrier biomarkers by *Lactobacillus delbrueckii* CIDCA 133 would be associated with its beneficial effects against 5-FU chemotherapy-induced intestinal mucositis.

## Materials and Methods

### Bacterial Strain Growth Conditions

The bacteria *Lactobacillus delbrueckii* CIDCA 133 belongs to CIDCA 133 center (Centro de Investigación y Desarrollo en Criotecnología de Alimentos, Universidad Nacional de La Plata, Argentina). The strain was grown on MRS broth (Man, Rogosa and Sharpe) (Kasvi, São José dos Pinhais, Brazil) for 18 h at 37°C. CIDCA 133 (5 × 10^7^ CFU/mL) dose was determined by growth curves using counting colony formation-unit (CFU) (de Jesus et al., 2021b).

### Animals and Ethics Statement

Conventional BALB/c mice (male, weight 20-24 g, six weeks old) were obtained from the Federal University of Minas Gerais (UFMG, Belo Horizonte, Brazil). Mice were kept in polycarbonate-ventilated cages in a controlled room with a temperature of 25 ± 2°C under a 12 h light/dark cycle, a standard chow diet, and *ad libitum* access to water for 24 h before experiments. All procedures were promptly approved by the Animal Experimentation Ethics Committee (CEUA-UFMG, number 66/2021) and were done according to the Brazilian Society of Sciences in Laboratory Animals (SBCAL) guidelines.

### Experimental Design

Mice were grouped into four experimental groups (*n* = 6): negative control (NC), probiotic (CIDCA 133), inflamed (5-FU), and inflamed and treated with probiotic (5-FU + CIDCA 133). Mice consumed MRS broth (NC and 5-FU group) or CIDCA 133 (5 × 10^7^ CFU/mL) (CIDCA 133 and 5-FU + CIDCA 133 group) by continuous feeding for 13 days. Bottles were changed every 24 h. Mice (5-FU and 5-FU + CIDCA 133 group) were inflamed intraperitoneally (*i.p.*) on the 10^th^ day with a single injection of 5-Fluorouracil (300 mg/kg) (Fauldfluor^®^, Libbs, São Paulo, Brazil) (de Jesus et al., 2019). Control groups (NC and CIDCA 133) received saline solution (NaCl 0.9%) injection. Seventy-two hours after mucositis induction, the animals were euthanized by anesthesia deepening [xylazine (16 mg/kg) and ketamine (80 mg/kg)] (Ceva, São Paulo, Brazil). Liquid and feed intake and body weight were evaluated daily before euthanasia. Blood, thymus, spleen, and ileum sections were collected for analysis.

### Histological Analysis

After euthanasia, the entire small intestine length was measured. Ileum sections were washed with PBS 0.1 M, rolled up, and fixed in a 10% buffered formaldehyde solution (Labsynth, São Paulo, Brazil). This material was embedded in paraffin, sectioned at 4 μm thick, stained with hematoxylin and eosin (HE) for scoring and morphometric analysis, and periodic acid-Schiff (PAS) for goblet cells count.

The histological inflammation score was determined as previously described by Soares et al. (2008). For morphometric analysis, ten field images of the ileum of each animal were captured using a BX41 optical microscope (Olympus, Tokyo, Japan). Twenty villus heights and crypt depth (magnification of 200 ×) and ten field/slides of goblet cells count (magnification 400 ×) were measured. These analyses were performed using *ImageJ* 1.51j.8 software (NIH, Bethesda, MD, United States).

### Inflammatory Cell Infiltration

Neutrophil and eosinophil recruitment in the intestinal mucosa was performed by myeloperoxidase (MPO) and eosinophil peroxidase (EPO) enzyme activities, respectively (Barroso et al., 2021). For this purpose, mice ileum sections were homogenized, centrifuged, and lysed by hypotonic solution with three cycles of freezing and thawing in liquid nitrogen. After centrifugation, the supernatant was used to quantify enzyme activities (colorimetric assay). The assay was read at an absorbance of 492 nm (EPO) and 450 nm (MPO) on a microplate spectrophotometer (Spectramax M3, Molecular Devices, LLC, Sunnyvale, CA, United States). The results are expressed as MPO or EPO arbitrary units (AU)/mg of tissue.

### Mice Ileum Relative Gene Expression Analysis

#### Total RNA Isolation

Mice ileum (1.0 cm) was collected and stored in RNAlater (Invitrogen, Carlsbad, CA, United States) at −20°C to preserve the samples. Total RNA isolation was carried out using an RNeasy Mini Kit (QIAGEN, Hilden, Germany), and residual DNA was degraded by DNAse I from the TURBO DNA-free™ Kit (Invitrogen, Carlsbad, CA, United States) according to the kit protocol. Complementary deoxyribonucleic acid (cDNA) synthesis was performed according to the manual instructions of the Applied Biosystems High-Capacity cDNA Reverse Transcription kit (Thermo Fisher, Waltham, MA, United States).

#### Quantitative PCR (qPCR)

qPCR was performed using PowerUp™ SYBR^®^ Green Master Mix (Thermo Fisher, Waltham, MA, United States) and the gene-specific primers for expression of Toll-like receptor 2 and 4 (*Tlr2, Tlr4*), myeloid differentiation primary response gene 88 (*Myd88*), nuclear factor NF-kappa-B p105 subunit (*Nfkb1*), tumor necrosis factor (*Tnf*), interleukin 1 beta (*Il1b*), interleukin 6 (*Il6*), interleukin 10 (*Il10*), mucin 2 (*Muc2*), claudin 1 and 2 (*Cldn1, Cldn2*), junctional adhesion molecule 1 (*F11r*), zonulin (*Hp*) and occludin (*Ocln*). The glyceraldehyde-3-phosphate dehydrogenase (*Gapdh*) gene was used as an endogenous reference (Table 1; Giulietti et al., 2001; Song et al., 2013; Volynets et al., 2016; Zheng et al., 2017; Chang et al., 2020). Amplification cycles (initial denaturation at 95°C for 10 min, 95°C for 15 seg, annealing/extension at 60°C for 1 min, 40 cycles followed by a dissociation stage for recording the melting curve) were performed using Applied Biosystems 7900HT Fast Real-Time PCR System. The expression levels were presented as fold change using the 2^–ΔΔCT^ method. Data are representative of two independent experiments.

**TABLE 1:** Quantitative polymerase chain reaction (qPCR) primers used in this study.

Gene	Primer Forward	Primer Reverse	References
*Gapdh*	TCACCACCATGGAGAAGGC	GCTAAGCAGTTGGTGGTGCA	Giulietti et al., 2001
*Tlr2*	ACAATAGAGGGAGACGCCTTT	AGTGTCTGGTAAGGATTTCCCAT	Chang et al., 2020
*Tlr4*	ATGGCATGGCTTACACCACC	GAGGCCATTTTTGTCTCCACA	Chang et al., 2020
*Myd88*	ATCGCTGTTCTTGAACCCTCG	CTCACGGTCTAACAAGGCCAG	Chang et al., 2020
*Nfkb1 (p105)*	GTGGAGGCATGTTCGGTAGTG	TCTTGGCACAATCTTTAGGGC	Zheng et al., 2017
*Il6*	GAGGATACCACTCCCAACAGACC	AAGTGCATCATCGTTGTTCATACA	Giulietti et al., 2001
*Il10*	GGTTGCCAAGCCTTATCGGA	ACCTGCTCCACTGCCTTGCT	Giulietti et al., 2001
*Tnf*	ACGTGGAACTGGCAGAAGAG	CTCCTCCACTTGGTGGTTTG	Song et al., 2013
*Il1b*	CTCCATGAGCTTTGTACAAGG	TGCTGATGTACCAGTTGGGG	Song et al., 2013
*Muc2*	GATGGCACCTACCTCGTTGT	GTCCTGGCACTTGTTGGAAT	Volynets et al., 2016
*Cldn1*	TCCTTGCTGAATCTGAACA	AGCCATCCACATCTTCTG	Volynets et al., 2016
*Cldn2*	GTCATCGCCCATCAGAAGAT	ACTGTTGGACAGGGAACCAG	Volynets et al., 2016
*Ocln*	ACTCCTCCAATGGACAAGTG	CCCCACCTGTCGTGTAGTCT	Volynets et al., 2016
*Hp*	CCACCTCTGTCCAGCTCTTC	CACCGGAGTGATGGTTTTCT	Volynets et al., 2016
*F11r*	CACCTTCTCATCCAGTGGCATC	CTCCACAGCATCCATGTGTGC	Volynets et al., 2016

#### Intestinal Permeability Evaluation

Mice received 0.1 mL of DTPA (diethylenetriaminepentaacetic acid) via gavage, labeled with 18.5 MBq 99mtechnetium on the last experimental day (de Barros et al., 2018). After four hours, total blood was collected without any stabilizer and measured the radioactivity level in an automated gamma radiation counter (PerkinElmer Wallac Wizard 1470-020 Gamma Counter, Waltham, MA, United States). Permeability was calculated by the percentage of DTPA administered dose per gram of blood (% ID/g), as follows: counts per min = (blood *cpm*/*cpm* of the administered dose) × 100.

#### Organ Index

To evaluate the toxic effects of 5-FU on lymphopoietic organs, splenic and thymus indices were carried out as previously described by Ren et al. (2019), as following: organ index = (weight of organ/body weight of the mice) × 100.

#### Statistical Analysis

The data normality was performed using the Shapiro-Wilk test. Data were evaluated by one-way ANOVA followed by Tukey’s *post hoc* test (parametric data) or by the Kruskal-Wallis test and post-tested by Dunn’s test (non-parametric data). The Mann-Whitney test analyzed food and liquid intake before and after 5-FU-inflammation induction. Spearman test was used for correlation analysis. All data were performed using GraphPad Prism 8.0 software (GraphPad Software, San Diego, CA, United States.), with a *p-value* < 0.05. The results are presented as mean ± standard deviation.

## Results

### *Lactobacillus delbrueckii* CIDCA 133 Not Stop Weight Loss, or Liquid and Food Intake in 5-FU-Treated Mice

**FIGURE 1:**
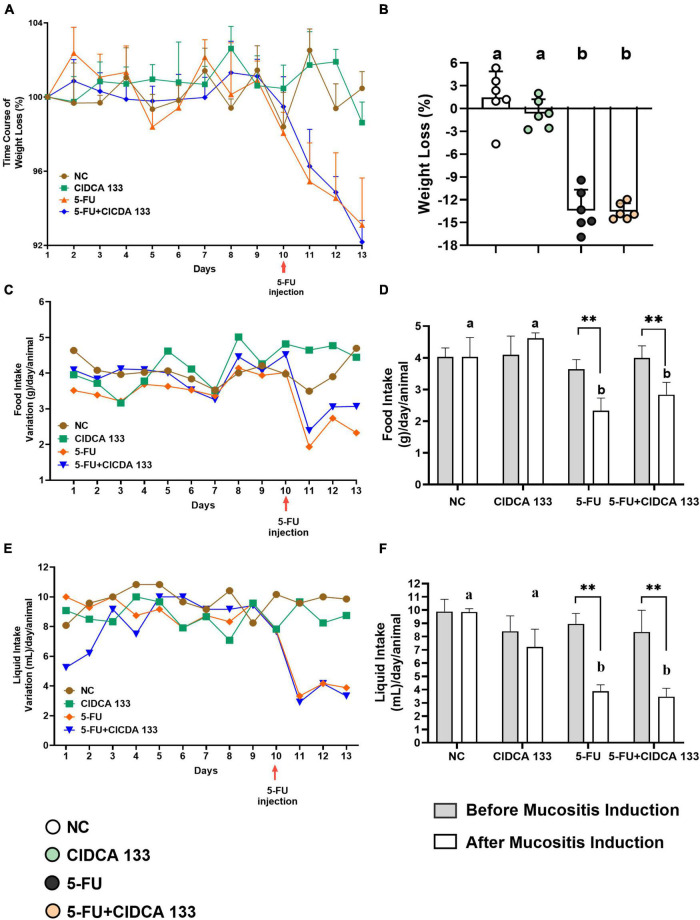
*Lactobacillus delbrueckii* CIDCA 133 effect on mice clinical parameters altered by 5-FU administration. **(A)** Daily body weight variation, **(B)** body weight loss, **(C)** daily food variation **(D)** food intake, **(E)** daily liquid variation **(F)** liquid intake. Different letters **(A,B)** and asterisks (*) indicate statistically significant differences (*p* < *0.05*) by ANOVA followed by Tukey’s post-test **(B)** and Mann-Whitney test **(D,F)**, respectively. Geometric symbols show animal numbers evaluated in each experimental group. *p < 0.05, **p < 0.01.

No abnormal clinical behavior or mortality was observed in mice before and after 5-FU inflammation induction. Body weight loss was also evaluated to check whether treatment with CIDCA 133 could relieve the symptoms of 5-FU-induced inflammation. The body weight variation in inflamed mice was observed 24 hours after the administration of the antineoplastic 5-FU (Figure 1A) when compared to control groups, with mice in the 5-FU group showing significant body weight loss (13.43 ± 2.76%) compared to the NC group (1.47 ± 3.41%) (*p* < *0.0001*) (Figure 1B). CIDCA 133 treatment (5-FU + CIDCA 133 group) was unable to mitigate 5-FU-induced body weight loss (13.54 ± 1.07%) (*p* = *0.998*) (Figure 1B).

Furthermore, the time course of the mice’s liquid and food intake was also evaluated (Figures 1C,E). As expected, after 10*^th^* the 5-FU group showed a significant reduction in the average food (2.33 ± 0.40 g) (Figure 1D) and liquid (3.79 ± 0.42 mL) (Figure 1F) consumption compared to the NC group (food intake: 4.03 ± 0.61 g; liquid intake: 9.81 ± 0.21 mL) (*p* < *0.01*). CIDCA 133 consumption (5-FU + CIDCA 133 group) did not improve these parameters (food intake: 2.83 ± 0.38 g; liquid intake: 3.47 ± 0.63 mL) (*p* = *0.49*) (Figures 1D,F).

### *Lactobacillus delbrueckii* CIDCA 133 Reduces 5-FU-Induced Inflammatory Cell Infiltration

The inflammatory infiltrates in the intestinal mucosa are one of the most common characteristics of 5-FU inflammation induction. In this study, we assessed the presence of ileum neutrophil and eosinophil infiltrates by detecting specific myeloperoxidase (MPO) and eosinophil peroxidase (EPO) enzyme activities, respectively.

**FIGURE 2:**
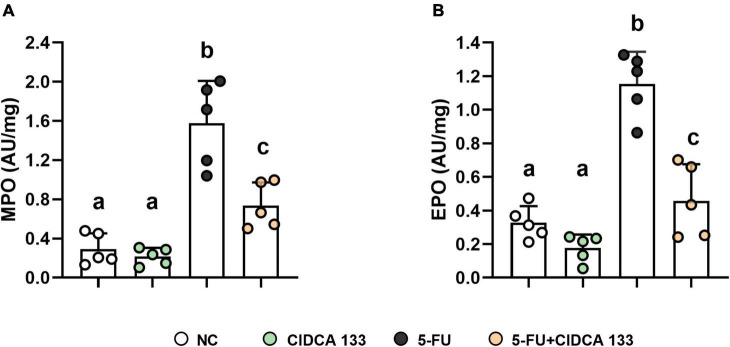
Ameliorative effect of *L. delbrueckii* CIDCA 133 on inflammatory cell infiltration. **(A)** myeloperoxidase, **(B)** eosinophil peroxidase. Different letters **(A,B)** indicate statistically significant differences (*p* < *0.05*) by ANOVA followed by Tukey’s post-test. Geometric symbols show animal numbers evaluated in each experimental group.

5-FU group showed an increased level of MPO (1.74 ± 0.57 AU/mg) (Figure 2A) and EPO (1.15 ± 0.19 AU/mg) (Figure 2B) activity when compared to the NC group (MPO: 0.34 ± 0.18 AU/mg; EPO: 0.32 ± 0.09 AU/mg) (*p* < *0.0001*). However, a reduction in neutrophil and eosinophil recruitment after CIDCA 133 (5-FU + CIDCA 133 group) administration (MPO: 0.73 ± 0.23 AU/mg; EPO: 0.45 ± 0.21 AU/mg) (*p* < *0.001*) (Figures 2A,B) was observed.

Regarding the toxic effects of 5-FU on lymphopoietic organs, administration of 5-FU (5-FU group) induced thymus (0.04 ± 0.008) and spleen (0.12 ± 0.009) atrophy compared to the NC group (thymus: 0.12 ± 0.03; spleen: 0.24 ± 0.01) (*p* < 0.001). However, CIDCA 133 consumption (5-FU + CIDCA 133 group) was not able to ameliorate this toxic effect (thymus: 0.03 ± 0.005, *p* = 0.89; spleen: 0.13 ± 0.005, *p* = 0.84).

### *Lactobacillus delbrueckii* CIDCA 133 Mitigates Intestinal Mucosa Damage Induced by 5-FU

**FIGURE 3:**
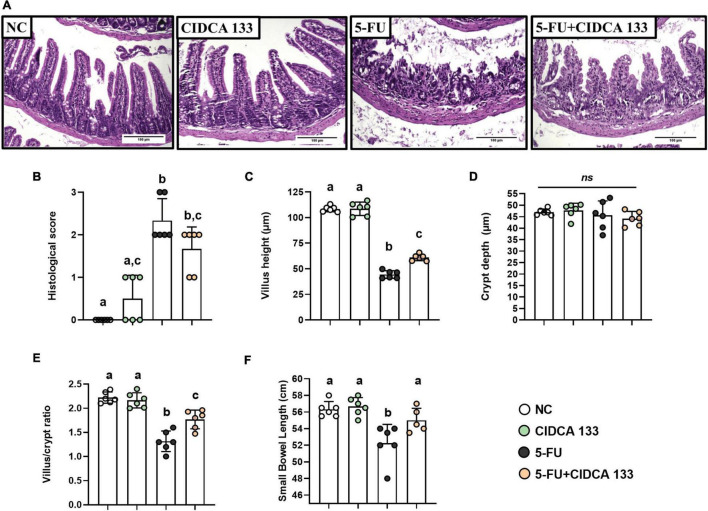
*Lactobacillus delbrueckii* CIDCA 133 mitigates 5-FU-induced epithelial architecture damage. **(A)** Histopathological images of mouse ileum (objective: × 20, scale 100 μm), **(B)** histopathological score, **(C)** villus height, **(D)** crypt depth, **(E)** villus height to crypt depth ratio, and **(F)** small intestine length. Different letters **(A–C)** indicate statistically significant differences (*p* < *0.05*) by Kruskal–Wallis test followed by Dunn’s *post hoc* test **(B)** or ANOVA followed by Tukey’s *post hoc* test **(C–F)**. Geometric symbols show animal numbers evaluated in each experimental group. *ns* indicates no significant difference (*p* > 0.05).

Regarding histopathological analysis, mice inflamed with 5-FU (5-FU) showed significant alterations in mucosal integrity, such as edema, villus shortening, crypt necrosis, goblet cell number reduction, and intense polymorphonuclear cells infiltration into the lamina propria and villi, compared to the NC group (Figure 3A). These results were correlated with the respective histopathological scores (Figure 3B).

The inflamed mice treated with CIDCA 133 (5-FU + CIDCA 133 group) showed a decrease in 5-FU-induced mucosal damage. Villus height preservation (Figure 3C), an increased villus height to crypt depth ratio (Figure 3E), and a reduction in inflammatory infiltration in the villus and lamina propria were observed histologically in the 5-FU + CIDCA 133 group compared to the 5-FU group. However, no significant difference in crypt depth was observed across groups (Figure 3D), and CIDCA 133 strain consumption (5-FU + CIDCA 133 group) did not improve the histological score (Figure 3B).

These above findings can be associated to preservation of intestine length. 5-FU administration (5-FU group) also reduced the small intestine length (52.75 ± 2.27 cm) compared to the control group (NC) (56.33 ± 0.93 cm) (*p* < *0.001*). However, treatment with CIDCA 133 (5-FU + CIDCA 133 group) prevented this shortening (55.00 ± 1.45 cm) at similar levels to the NC group (*p* < *0.05*) (Figure 3F).

### *Lactobacillus delbrueckii* CIDCA 133 Modulates Gene Expression of the TLR2/4/Myd88/NF-κB Signaling Pathway

**FIGURE 4:**
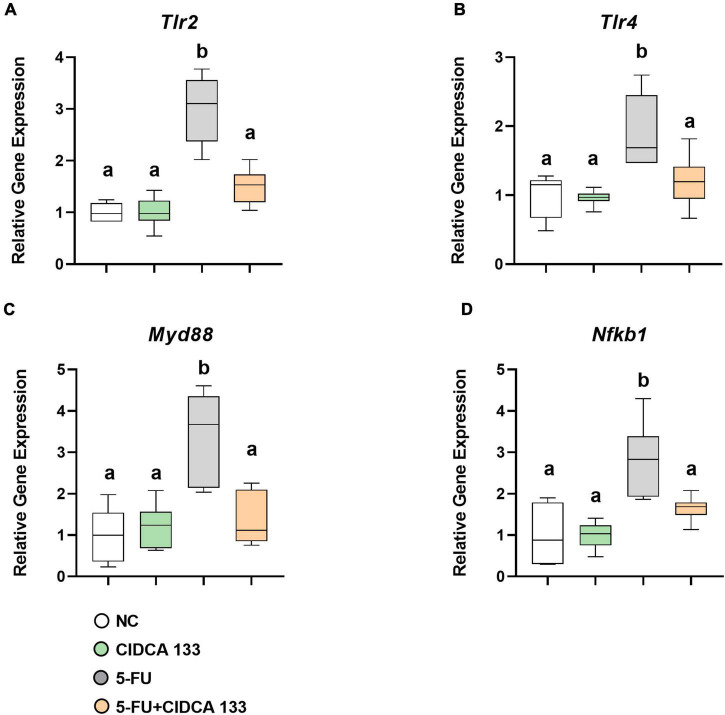
*Lactobacillus delbrueckii* CIDCA 133 downregulates the gene expression of markers involved in NF-κB signaling pathway activation induced by 5-FU. **(A)** *Tlr2*, **(B)** *Tlr4*, **(C)** *Myd88*, and **(D)** *Nfkb1*. Different letters **(A,B)** indicate statistically significant differences (*p* < *0.05*) by ANOVA followed by Tukey’s post-test.

Mice in the 5-FU group displayed significantly increased mRNA expression of *Tlr2* (2.99 ± 0.64; *p* < *0.0001*) (Figure 4A), *Tlr4* (1.90 ± 0.52; *p* < *0.001*) (Figure 4B), *Myd88* (3.33 ± 1.14; *p* < *0.0001*) (Figure 4C), and *Nfkb1* (2.80 ± 0.88; *p* < *0.0001*) (Figure 4D) compared to the NC group. Compared to the 5-FU-treated group, the mRNA expression profiles of *Tlr2* (1.50 ± 0.33; *p* < *0.0001*) (Figure 4A), *Tlr4* (1.19 ± 0.37; *p* < *0.01*) (Figure 4B), *Myd88* (1.36 ± 0.62; *p* < *0.001*) (Figure 4C), and *Nfkb1* (p105) (1.64 ± 0.30; *p* < *0.01*) (Figure 4D) were significantly reduced after CIDCA 133 consumption (5-FU + CIDCA 133).

### *Lactobacillus delbrueckii* CIDCA 133 Downregulates Proinflammatory Cytokines and Upregulates *Il10* Gene Expression

**FIGURE 5:**
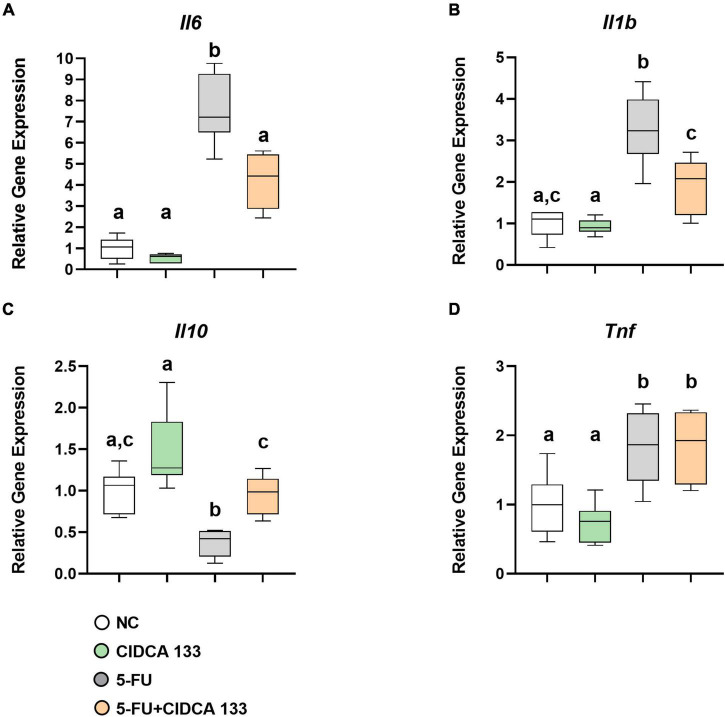
*Lactobacillus delbrueckii* CIDCA 133 downregulates 5-FU-induced proinflammatory cytokine gene expression. **(A)** *Il6* **(B)** *Il1b*, **(C)** *Il10*, and **(D)** *Tnf*. Different letters **(A–C)** indicate statistically significant differences (*p* < *0.05*) by ANOVA followed by Tukey’s post-test.

Mice in the 5-FU group exhibited a significant increased mRNA expression of *Il6* (7.57 ± 1.63; *p* < *0.0001*) (Figure 5A), *Il1b* (3.26 ± 0.83; *p* < *0.0001*) (Figure 5B) and *Tnf* (1.82 ± 0.52; *p* < *0.05*) (Figure 5D) and a downregulation of *Il10* (0.37 ± 0.15; *p* < *0.01*) (Figure 5C) compared to the NC group. After CIDCA 133 consumption (5-FU + CIDCA 133 group), downregulation of *Il6* (4.21 ± 1.29; *p* < *0.001*) (Figure 5A) and *Il1b* (1.191 ± 0.68; *p* < *0.001*) (Figure 5B) and upregulation of *Il10* (0.95 ± 0.24; *p* < *0.01*) (Figure 5C) mRNA expression were observed compared to the 5-FU group. No difference in *Tnf* (1.84 ± 0.49; *p* = *0.99*) (Figure 5D) gene expression was observed between the 5-FU + CIDCA 133 group and the 5-FU group.

### *Lactobacillus delbrueckii* CIDCA 133 Reduces 5-FU-Induced Goblet Cell Depletion and Upregulates *Muc2* Gene Expression

**FIGURE 6:**
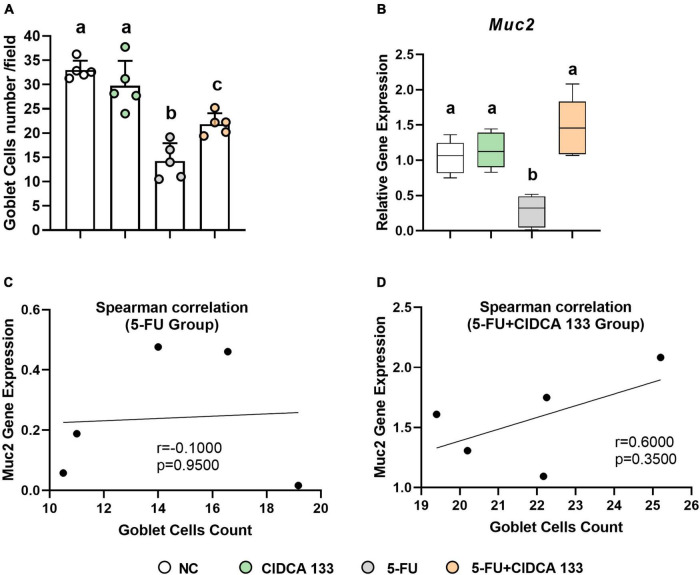
*Lactobacillus delbrueckii* CIDCA 133 reduces 5-FU-induced goblet cell depletion and upregulates *Muc2* gene expression. **(A)** Goblet cell number and **(B)** relative gene expression of mucin 2 (*Muc2)* **(C,D)** Spearman correlation between goblet cell count and *Muc2* gene expression. Different letters **(A–C)** indicate statistically significant differences (*p* < *0.05*) by ANOVA followed by Tukey’s post-test.

Significant goblet cell number reduction was also observed in the 5-FU-treated (5-FU group) ileum-inflamed mice (14.25 ± 3.68 cell/field) compared to the NC group (32.99 ± 1.91 cell/field) (*p* < *0.0001*), but treatment with CIDCA 133 (5-FU + CIDCA 133) reduced the loss of goblet cells (21.84 ± 2.24 cell/field) (*p* < *0.05*) (Figure 6A). A downregulation of *Muc2* gene expression was also exhibited in the inflamed ileum of the 5-FU mice (5-FU group) (0.28 ± 0.22) (*p* < *0.001*) and its upregulation after treatment with CIDCA 133 (5-FU + CIDCA 133 group) (1.48 ± 0.40) (*p* < *0.0001*) (Figure 6B) was observed. No positive correlation was observed between villus’ goblet cells count and *Muc2* gene expression among inflamed (5-FU group) (*r* = -0.1000, *p* = 0.9500) (Figure 6C) and treated mice (5-FU + CIDCA 133 group) (*r* = 0.6000, *p* = 0.3500) (Figure 6D).

### *Lactobacillus delbrueckii* CIDCA 133 Ameliorates 5-FU-Induced Increased Intestinal Permeability and Upregulates Tight Junction Gene Expression

**FIGURE 7:**
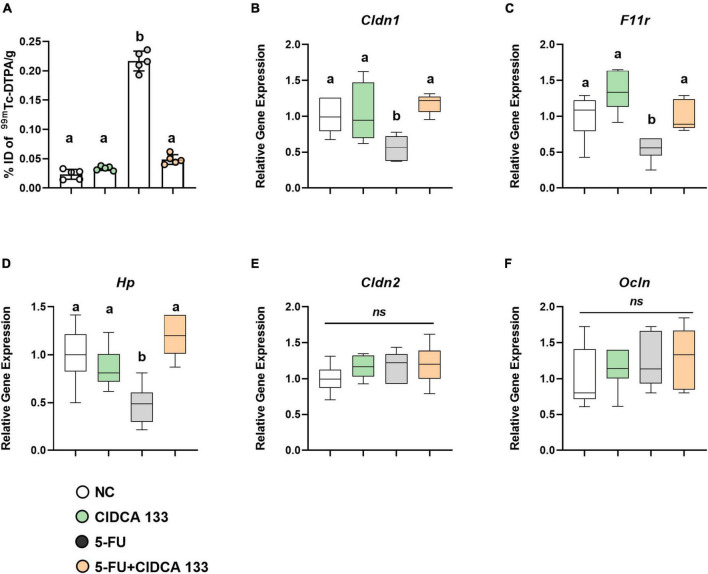
*Lactobacillus delbrueckii* CIDCA 133 reduces 5-FU-induced intestinal permeability increase and upregulates tight junction protein gene expression. **(A)** Intestinal permeability, **(B–F)** Relative gene expression of tight junction proteins. Different letters **(A–C)** indicate statistical differences by ANOVA followed by Tukey’s post-test (*p* < *0.05*).

Intestinal permeability was evaluated by measuring the radioactivity uptake in the blood after oral administration of radiolabeled diethylenetriamine-pentaacetate (^*99m*^Tc-DTPA). As expected, 5-FU administration (5-FU group) significantly increased intestinal permeability in treated mice (0.21 ± 0.01% ID/g) compared to the NC group (0.02 ± 0.00% ID/g) (*p* < *0.0001*). However, treatment with CIDCA 133 (5-FU + CIDCA 133 group) significantly reduced 5-FU-induced intestinal permeability (0.04 ± 0.01% ID/g; *p* < *0.0001*) (Figure 7A).

Gene expression of tight junction proteins was also measured. Mice inflamed with 5-FU (5-FU group) exhibited a significant reduced mRNA expression of tight junction proteins *Cldn1* (0.56 ± 0.17; *p* < *0.05*) (Figure 7B), *Fr11* (0.54 ± 0.16; *p* < *0.05*) (Figure 7C), and *Hp* (0.47 ± 0.20; *p* < *0.01*) (Figure 7D) compared to the NC group. The mRNA expression of *Cldn1* (1.17 ± 0.13), *Fr11* (0.99 ± 0.20) and *Hp* (1.19 ± 0.21) was significantly upregulated after CIDCA 133 consumption (5-FU + CIDCA 133 group) compared to the 5-FU group (*p* < *0.01*). No differences in *Cldn2* (1.20 ± 0.27) (Figure 7E) and *Ocln* (1.29 ± 0.44) (Figure 7F) gene expression were observed across the groups (*p* > *0.05*).

## Discussion

5-Fluorouracil (5-FU) is a commonly used antineoplastic drug that shows intestinal mucositis as one of its main side effects. This gastrointestinal disorder is characterized by inflammation and ulceration of the intestinal epithelium (Sonis, 2004) and is a limiting factor in oncology therapy success.

Probiotic consumption has been considered a promising therapeutic option to ameliorate the inflammatory and epithelial damage induced by 5-FU administration due to their reported anti-inflammatory effects (Batista et al., 2020; Cristofori et al., 2021). The protective effect of *L. delbrueckii* CIDCA 133 on 5-FU-induced epithelial damage was previously demonstrated using a fermented milk formulation (de Jesus et al., 2019). In this study, we investigated whether the beneficial effect of *L. delbrueckii* CIDCA 133 on damage caused by 5-FU-induced intestinal mucosal would be associated with the ability of this probiotic strain to regulate the gene expression of inflammatory and epithelial barrier markers.

Due to 5-FU cytotoxicity, severe epithelial damage has been reported as one of the most critical features of mucositis pathobiology. This damage includes epithelial architecture, mucosal barrier integrity alteration, goblet cell depletion, villus shortening, intestinal permeability, and increased polymorphonuclear cell infiltration. All these parameters were evaluated in this study to assess the ameliorative and anti-inflammatory activity of the CIDCA 133 strain. Although CIDCA 133 treatment did not improve the histological score, the strain was able to reduce the tissue damage and preserve villus height induced by 5-FU, corroborating with previous studies that had demonstrated the protective effect of other probiotics in the intestinal architecture of inflamed mice with 5-FU (Yeung et al., 2015; Kato et al., 2017; Oh et al., 2018).

It is essential to highlight that in addition to the damage reported at the ileal level, we also observed that 5-FU affected the whole digestive tract and other organs’ functions. This finding can be demonstrated by shortening the small intestine length after administering the antineoplastic agent. 5-FU promoted intestinal shortening, and mucosal destruction led to reduced nutrient absorption, which might correlate with body weight loss observed during mucositis (Vieira et al., 2012; Maioli et al., 2014; Kato et al., 2017; de Barros et al., 2018).

Our study shows that consumption of CIDCA 133 reduced the shortening of the small intestine but did not improve food and hydric intake, and body weight loss. Similar results were reported by Maioli et al. (2014) and Antunes et al. (2016) in animals with mucositis induced by 5-FU. The researchers observed that their experimental treatment did not influence food intake and weight loss. These results reinforce how intestinal mucositis, as a side effect of 5-FU administration, may become a severe problem in chemotherapy. In addition, unlike what was observed, the beneficial effect of milk fermented by CIDCA 133 on body weight loss and histopathological score in inflamed mice with 5-FU has been previously reported by us (de Jesus et al., 2019), demonstrating that dairy formulation with this probiotic seems to be more promising in the clinical manifestations of 5-FU administration-derived mucositis. One possible explanation for this outcome is that dairy formulations possess nutraceutical compounds (*e.g.*, amino acid, fibers) as well as probiotic-derived bioactive metabolites (*e.g.*, SCFA) (Galdino et al., 2018; Trindade et al., 2018), making them more nutritious and beneficial than MRS culture broth. Furthermore, despite improving the probiotic’s inflammatory parameters, the positive effects on liquid and food intake, and body weight may be more prominent with the intestinal healing progresses, ameliorating the gastrointestinal dysmotility (Soares et al., 2008), increasing the area of nutrient absorption, and consequently improving the mice’s clinical status (de Jesus et al., 2019). It is also interesting to point out that although probiotics demonstrate multiple beneficial effects, a single strain does not seem to be clinically sufficient, so it is proposed that the combination of several probiotic strains may be most effective (Prisciandaro et al., 2011a; Yeung et al., 2015).

The production of several inflammatory markers, such as proinflammatory cytokines (IL-6, TNF-α, IL-1β, IFN-γ, CXCL1), via exacerbated activation of the TLR2/4/Myd88/NF-κB signaling pathway, is another feature caused by the administration of 5-FU (Chang et al., 2012; Li et al., 2017; Justino et al., 2020). Probiotics can modulate the host immune response through their interaction with intestinal epithelial cells via Toll-like receptors (TLRs) and regulate the balance between the T helper type 1 and 2 (Th1 and Th2) responses induced by NF-κB activation (Kawai and Akira, 2007; Mukherjee et al., 2016). Therefore, we also investigated the effects of CIDCA 133 consumption and its impact on the gene expression associated with immune system activation. We observed a decrease in the relative gene expression of *Tlr2*, *Tlr4*, *Myd88*, and *Nfkb1* after CIDCA 133 strain consumption by inflamed mice, while the mRNA expression of proinflammatory cytokines (*Il6* and *Il1b*) and regulatory cytokine *Il10* were down- and upregulated, respectively. Regarding TNF cytokine, its gene expression was upregulated after 5-FU administration, and transcript levels were maintained after consumption of CIDCA 133. TNF is a key factor in inflammatory diseases. However, it has been demonstrated that epithelial-derived TNF-α can stimulate epithelial cell proliferation, a mechanism used by probiotics to promote intestinal epithelial barrier regeneration and improve the innate immune response (Pagnini et al., 2010; Giorgetti et al., 2015). This property may be a mechanism used by CIDCA 133 in its beneficial effect against 5-FU epithelial damage.

The balance of pro-and anti-inflammatory cytokines is fundamental to control the exacerbation of intestinal damage in 5-FU-induced mucositis. Upregulation of *Il10* in mice inflamed with 5-FU and treated with CIDCA 133 evidenced the anti-inflammatory effects that this strain can provide. This finding corroborates de Jesus et al. (2021b), who showed that CIDCA 133 could modulate inflammatory responses possibly by controlling NF-κB signaling pathway activation through upregulation of immunoregulatory molecules such as *Il10* and *Tgfb1* to maintain intestinal homeostasis.

Our results are supported by other studies in which administration of probiotics, such as *Saccharomyces boulardii* CNCM I-745 (Justino et al., 2020), *Lactobacillus acidophilus* (Justino et al., 2015), or *Lacticaseibacillus casei* (Yeung et al., 2015), also improved the inflammatory and functional aspects of intestinal inflammation induced by 5-FU through the modulation of the expression of *Tlr2, Tlr4, Myd88, Nf-*κ*B*, and cytokines *Il6, Il1b, Il10*, and *Ifng*. It should be noted that not all probiotics showed anti-inflammatory effects in 5-FU-induced epithelial damage (Prisciandaro et al., 2011b; Maioli et al., 2014). Despite sharing similar probiotic features (Plaza-Diaz et al., 2019), the mechanisms used in a 5-FU inflammation context may be different from one strain/specie to another, and this may be related to the probiotic dose used, the antineoplastic agents type and experimental protocols for inducing mucositis, as well as the biotic form as beneficial microorganisms are administrated (viable, inactivated or their secreted products) (Chang et al., 2012; Yeung et al., 2015; Batista et al., 2020; Wu et al., 2021).

The intestinal mucosa destruction and production of proinflammatory cytokines induced 5-FU cytotoxicity, resulting in the recruitment of eosinophils and neutrophils into the lamina propria. This study shows increased activities of MPO and EPO in 5-FU-inflamed mice, which was reduced after administration of CIDCA 133. The beneficial effect of this strain on the recruitment of eosinophils and neutrophils to the intestinal mucosa reduced the oxidative stress and production of proinflammatory cytokines induced by these cells. Consequently, it prevented the exacerbation of tissue damage and the development of ulcers. These findings are consistent with other studies that also observed beneficial effects of different probiotic microorganisms in decreasing inflammatory cell infiltrate induced by 5-FU administration, such as *Bifidobacterium sp*. (Kato et al., 2017; Quaresma et al., 2020), *Lactobacillus acidophilus* (Oh et al., 2018) *Lacticaseibacillus rhamnosus* (Trindade et al., 2021) and *Rhodotorula mucilaginosa* (Coutinho et al., 2021).

Polymorphonuclear cell infiltration is also associated with an increase in intestinal permeability (IP), probably as a result of reactive oxygen species (ROS) production by these cells, villous atrophy, and reduced expression of tight junction proteins (van Vliet et al., 2010; Li et al., 2017). In this study, IP was studied by measuring blood radioactivity after oral intake of ^*99m*^Tc-DTPA, a hydrophilic macromolecule that rarely crosses the intestinal barrier under healthy conditions, making it a good radiotracer marker for measuring IP (Andrade et al., 2015). CIDCA 133 consumption was able to prevent intestinal permeability induced by 5-FU significantly. The permeability was decreased due to upregulation of tight junctions (*Cldn1, F11r*, and *Hp*) and *Muc2* gene expression, reduction in mucus-producing goblet cell number loss, and villus preservation by the strain. These results agree with other studies that also showed beneficial effects of probiotics in attenuating 5-FU-induced intestinal permeability (Tang et al., 2017; Trindade et al., 2018; Porto et al., 2019) and goblet cell number loss (Yeung et al., 2015; Kato et al., 2017; de Jesus et al., 2019).

Reactive oxygen species production by inflammatory cells promotes intestinal cell apoptosis (Soares et al., 2008). On the basis of this information, we suggest CIDCA 133 can maintain the maturation of mucus-producing goblet cells and tight junctions gene expression by reducing apoptosis of epithelial cells resulting from inflammatory pathways activation, via direct contact of its bacterial cells components (*e.g*., surface or extracellular proteins, SCFA) with intestinal cells receptors (*e.g.*, TLR2/TLR4; GPR43) (Karczewski et al., 2010; van Vliet et al., 2010; Wang et al., 2018; Liu et al., 2020; Rose et al., 2021). This process would reduce the inflammatory cells infiltrate and their oxidative stress generated on the intestinal mucosa, thus preserving the function and structure of intestinal epithelial components, such as tight-junction proteins, goblet cells, and enterocytes. Furthermore, our results showed that although there was no difference between the groups related to crypt depth, there was an improvement in the crypt-villus ratio after consumption of CIDCA 133, demonstrating that possibly villus height improvement may be associated with the migration of enterocytes, produced by the crypt stem cells, to repair the intestinal epithelium by replacing the dead cells (Yeung et al., 2015). This, therefore, reflects the increase in the number of goblet cells present in the villi, as observed in our study.

Stringer et al. (2009) suggested that rapid mucin secretion in the small intestine due to 5-FU administration occurs primarily in the crypts, mainly caused by enteric neurotransmitters acting on epithelial cells, including goblet cells (Stringer et al., 2009). Furthermore, studies demonstrate that the maturation of goblet cells results from epithelial activation of TLR2/TLR4 (Podolsky et al., 2009; Dheer et al., 2016). On the other hand, TLR activation is also attributed to dysbiotic commensal microbiota (Li et al., 2017). Based on this information, we suggest that CIDCA 133 can upregulated mucin 2 as a mechanism to control the dysbiotic microbiota and maintain intestinal homeostasis. Altogether, these above mechanisms may lead to translocation inhibition of pathogenic microorganisms and toxins into the lamina propria, thereby attenuating an exacerbated inflammatory response (Sonis, 2004; Krishna Rao and Samak, 2013; Maioli et al., 2014), and, thus, reinforcing the importance of this strain in maintaining the epithelial barrier, as previously demonstrated by de Jesus et al. (2019) and de Jesus et al. (2021a).

The ameliorative effect of CIDCA 133 has already been demonstrated by our research group using a dairy formulation, which so far has been shown to be the best matrix for the beneficial effects of CIDCA 133 when compared to some results of our work. However, our findings showed the main molecular pathways used by this probiotic strain to promote its beneficial effects, which have not been reported in previous studies.

## Conclusion

Our results reinforce that *Lactobacillus delbru*eckii CIDCA 133 strain consumption improved mucosal damage in mice undergoing chemotherapy with 5-FU, demonstrating to be a promising adjuvant therapeutic strategy to attenuate 5-FU-induced intestinal mucositis. Furthermore, our work suggests that this strain can re-establish intestinal mucosa homeostasis involving different molecular mechanisms through modulation of NF-κB activation, proinflammatory cytokines, and tight junction proteins expression. Therefore, more studies will be performed to fully explain the mechanisms used for this strain on intestinal inflammation context, reinforcing its use as a probiotic.

## Data Availability Statement

The raw data supporting the conclusions of this article will be made available by the authors, without undue reservation.

## Ethics Statement

The animal study was reviewed and approved by Local Animal Experimental Ethics Committee of the Federal University of Minas Gerais (CEUA-UFMG).

## Author Contributions

FB, PM-A, and LJ: conceptualization. FB, LJ, VB, MD, KV, NC-R, FM, SF, and VC: methodology. LJ, FB, and EF: formal analysis and investigation. FB, LJ, JL, and TS: writing-original draft preparation. AB, DB, and VA: writing-review and editing. All authors read and approved the final manuscript.

## Conflict of Interest

The authors declare that the research was conducted in the absence of any commercial or financial relationships that could be construed as a potential conflict of interest.

## Publisher’s Note

All claims expressed in this article are solely those of the authors and do not necessarily represent those of their affiliated organizations, or those of the publisher, the editors and the reviewers. Any product that may be evaluated in this article, or claim that may be made by its manufacturer, is not guaranteed or endorsed by the publisher.
